# A novel *ABCA12* pathologic variant identified in an Ecuadorian harlequin ichthyosis patient: A step forward in genotype‐phenotype correlations

**DOI:** 10.1002/mgg3.608

**Published:** 2019-03-27

**Authors:** Martha Montalván‐Suárez, Uxia Saraiva Esperón‐Moldes, Laura Rodríguez‐Pazos, Andrés Ordóñez‐Ugalde, Fernanda Moscoso, Nora Ugalde‐Noritz, Luis Santomé, Laura Fachal, Daniel Tettamanti‐Miranda, Juan Carlos Ruiz, Manuel Ginarte, Ana Vega

**Affiliations:** ^1^ Sistema de Investigación y Desarrollo SINDE, Universidad Católica de Santiago de Guayaquil and Universidad de Guayaquil Guayaquil Ecuador; ^2^ Fundación Pública Galega de Medicina Xenómica‐SERGAS, Grupo de Medicina Xenómica‐USC, CIBERER, IDIS Santiago de Compostela Spain; ^3^ Departamento de Ciencias Forenses Anatomía Patolóxica, Xinecoloxía, Obstetricia e Pediatría, Universidade de Santiago de Compostela Santiago de Compostela Spain; ^4^ Servicio de Dermatología del Complejo Hospitalario Universitario de Vigo Vigo Spain; ^5^ Laboratorio Biomolecular Cuenca Ecuador; ^6^ Unidad de Genética y Molecular del Hospital de Especialidades José Carrasco Arteaga Cuenca Ecuador; ^7^ Universidad Espíritu Santo and Hospital Luis Vernaza Guayaquil Ecuador; ^8^ Instituto de Biomedicina Universidad Católica de Santiago de Guayaquil and Centro de Investigación, Universidad Espíritu Santo Guayaquil Ecuador; ^9^ Servicio de Dermatología del Complexo Hospitalario Universitario de Santiago, Facultad de Medicina Santiago de Compostela Spain

**Keywords:** *ABCA12* gene, Autosomal recessive congenital ichthyoses (ARCI), congenital ichthyosiform erythroderma (CIE), harlequin ichthyosis (HI), lamellar ichthyosis (LI), splice‐site pathogenic variant

## Abstract

**Background:**

Autosomal recessive congenital ichthyoses (ARCI) have been associated with different phenotypes including: harlequin ichthyosis (HI), congenital ichthyosiform erythroderma (CIE), and lamellar ichthyosis (LI). While pathogenic variants in all ARCI genes are associated with LI and CIE phenotypes, the unique gene associated with HI is *ABCA12*. In HI, the most severe ARCI form, pathogenic variants in both *ABCA12* gene alleles usually have a severe impact on protein function. The presence of at least one non‐truncating variant frequently causes a less severe congenital ichthyosis phenotype (LI and CIE).

**Methods:**

We report the case of a 4‐year‐old Ecuadorian boy with a severe skin disease. Genetic diagnosis was performed by NGS. In silico predictions were performed using Alamut software v2.11. A review of the literature was carried out to identify all patients carrying *ABCA12* splice‐site and missense variants, and to explore their genotype‐phenotype correlations.

**Results:**

Genetic testing revealed a nonsense substitution, p.(Arg2204*), and a new missense variant, p.(Val1927Leu), in the *ABCA12* gene. After performing in silico analysis and a comprehensive review of the literature, we conclude that p.(Val1927Leu) affects a well conserved residue which could either disturb the protein function or alter the splicing process, both alternatives could explain the severe phenotype of our patient.

**Conclusion:**

This case expands the spectrum of *ABCA12* reported disease‐causing variants which is important to unravel genotype‐phenotype correlations and highlights the importance of missense variants in the development of HI.

## 1 INTRODUCTION

Autosomal recessive congenital ichthyoses (ARCIs) are a heterogeneous disease that can present with a wide range of phenotypes including harlequin ichthyosis, (HI), congenital ichthyosiform erythroderma (CIE), and lamellar ichthyosis (LI). HI is the most severe form of congenital ichthyoses (Fischer, 2009; Oji et al., 2010). Neonates are born encased in a thick skin that not only restricts their movements, but also distorts their facial features, averting their lips and eyelids. Although newborns frequently die within the first few days of life, some of them survive, and their skin eventually resembles severe CIE or LI. ARCI is a genetically heterogeneous condition that can be caused by pathogenic variants in at least 12 genes including *TGM1 *(OMIM #190195), *ABCA12 *(OMIM #607800), *NIPAL4 *(OMIM #609383), *CYP4F22 *(OMIM #611495), *ALOX12B *(OMIM #603741), *ALOXE3 *(OMIM #607206), *LIPN *(OMIM #613924), *PNPLA1 *(OMIM #612121), *CERS3 *(OMIM #615276), *SDR9C7 *(OMIM #609769), *SULT2B1 *(OMIM #604125), and *CASP14 *(OMIM #605848) (Fischer, 2009; Grall et al., 2012; Heinz et al., 2017; Kirchmeier, Zimmer, Bouadjar, Rösler, & Fischer, 2017; Lefèvre et al., 2003, 2006; Radner et al., 2013; Shigehara et al., 2016).

*ABCA12* encodes a keratinocyte‐associated lipid transporter. Pathogenic variants in *ABCA12* are known to cause the three major phenotypes of ARCI: HI, LI, and CIE. Genotype‐phenotype correlations have been established in *ABCA12* associated disorders: homozygotes or compound heterozygotes with truncating *ABCA12* variants generally lead to an HI phenotype while homozygous missense variants usually cause a milder phenotype (Akiyama, 2010).

Here we report a boy suffering from HI with compound heterozygous disease‐causing variants in *ABCA12*, one truncating mutation: nonsense variant c.6610C>T, p.(Arg2204*), and a novel missense variant, not previously reported: c.5779G>T, p.(Val1927Leu). The location of the new disease‐causing variant (first nucleotide of exon 39) suggests it can potentially alter the splicing process. In order to understand the effect of *ABCA12* splice‐site and missense pathogenic variants, a literature search was performed.

## 2 CASE REPORT

The patient is a 4‐year‐old boy who was the third child of apparently non consanguineous parents from Manta, Manabí, Ecuador. There was no family history of congenital ichthyosis. Gestational age was approximately 7 months. After delivery the baby was placed in an incubator, where he spent one month. His mother mentioned that at birth he had several characteristics related to a harlequin fetus: thick large fissures over the whole body, flattened nose and ears, respiratory distress and feeding difficulties that required supplemental tube feeding; although he suffered from these complications he was able to breastfeed when he left the hospital. He also had toe blisters soon after birth that converted in toes synechia, affecting his gait. During the neonatal period the patient only received topical treatments.

**Figure 1:**
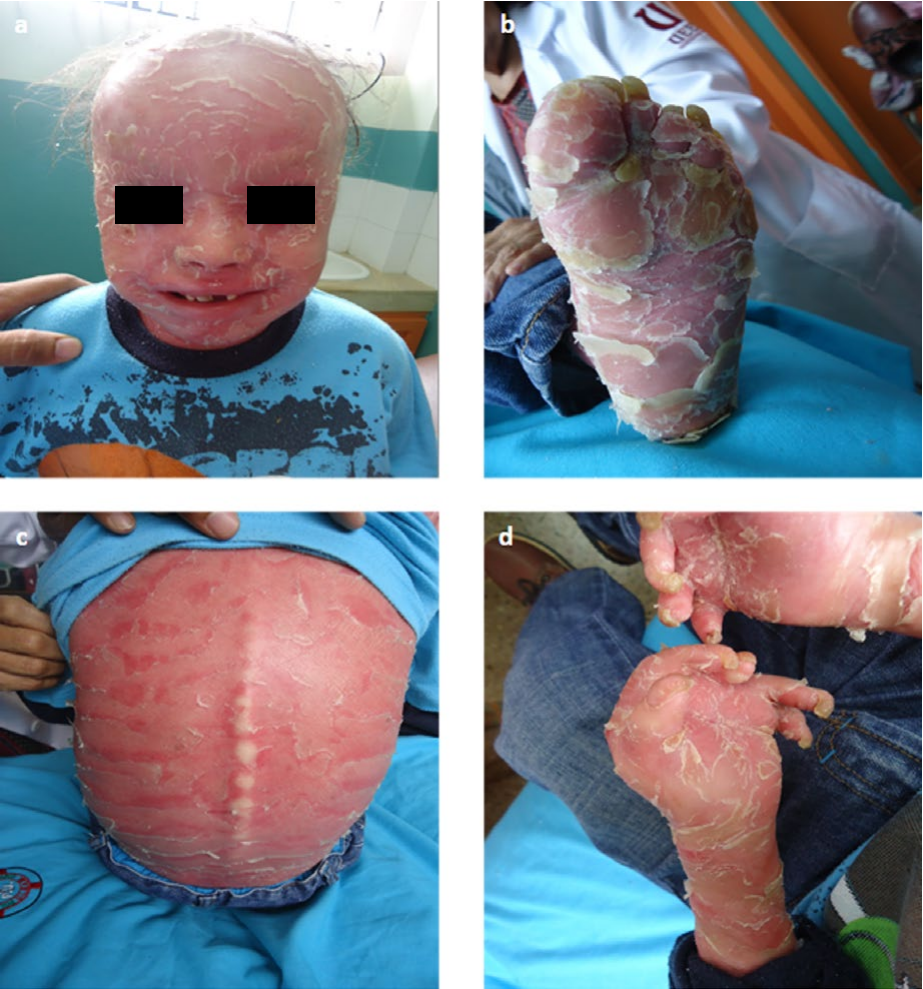
Clinical features of the patient: (a) Severe ectropion and almost complete alopecia, (b) Nail deformities and palmoplantar hyperkeratosis of the feet, (c) Patient's back showing large scales on an erythrodermic background, (d) Upper extremities severely affected. Retraction at finger joints

Physical examination revealed: ectropion, eclabium, nasal hypoplasia, rudimentary external ears, dental hypoplasia, erythema, inflammation of the gums, and almost complete alopecia (Figure 1a). He presented generalized scales on an erythrodermal background with abundant fissures (Figure 1c). Upper‐extremities showed a high degree of retraction at finger joints, giving a claw hand aspect (Figure 1d). There were nail deformities, abundant fissures in bending sites and palmoplantar hyperkeratosis (Figure 1b). During the clinical examination the patient showed sensitivity and irritability, due to the pain caused by the fissures, when he moved. After obtaining informed consent, blood extraction was performed in the affected child, his parents, and his healthy sisters. Genomic DNA was isolated from peripheral blood cells using standard procedures in the Biomolecular Laboratory located in Cuenca, Ecuador and sent to the Fundación Pública Galega de Medicina Xenómica in Spain, where genetic diagnosis was carried out. Ethical approval was obtained and all research was performed in accordance with the principles of the Declaration of Helsinki. Three micrograms of patient's genomic DNA were enriched using SureSelect (Agilent Technologies) following the manufacturer's protocol. The target resequencing library was then sequenced on a SOLiD 5500xl (Life Technologies). Color space reads were mapped to the GRCh37/hg19 reference genome using LifeScope software version 2.5.1 (Life Technologies). Finally, variants were identified using GATK version 2.1 (Genome Analysis Toolkit, Broad Institute) and LifeScope version 2.5.1 and annotated with ANNOVAR version 2012Mar08. In silico prediction of potential variant effects on splicing were computed by using MaxEnt, NNSPLICE, and Splice Site Finder. Missense prediction analyses were performed by using Align GV‐GD, SIFT, and Mutation Taster. All these algorithms are integrated in the Alamut® Visual 2.11 software (Interactive Biosoftware, Rouen, France). The review of the existing literature on splice‐site and missense *ABCA12 *mutations was carried out by taking into consideration each of all carrier patients reported to date.

## 3 RESULTS

**Figure 2:**
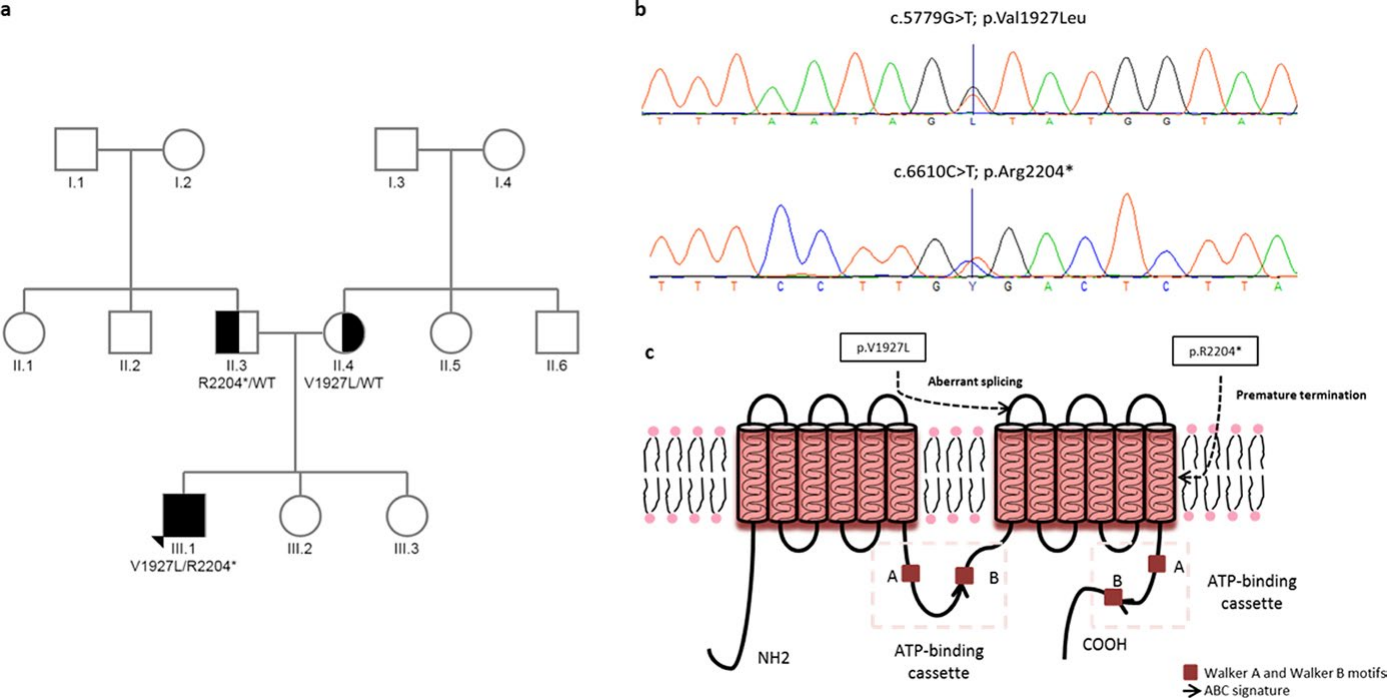
Pedigree of patient's family, electropherograms of both mutations and their location in the ABCA12 protein. (a) The patient (III:1) was a compound heterozygote for two *ABCA12* mutations, a novel splice site mutation p.(Val1927Leu) and the nonsense mutation p.(Arg2204*). His parents were heterozygous carriers, (b) Electropherograms of both heterozygous mutations identified in the proband, (c) Representation of the ABCA12 protein structure and the location of the two identified mutations

A total of 18 variants were identified in the patient's *ABCA12* gene (NM 173076.2, NP_775099). Sixteen were filtered out while two putative *ABCA12* variants in heterozygous state were prioritized by its location in the gene, the type of change they originated, and the frequency in 1000G project (http://www.1000genomes.org/): (a) a transition from C to T in exon 44: c.6610C>T; p.(Arg2204*) that leads to a nonsense substitution; it is located in the second transmembrane domain of the ABCA12 protein (Figure 2b,c). It has been previously identified in homozygous state in an African American patient that was born at 36 weeks of gestation, and died at 6 months of age from septicemia (Kelsell et al., 2005), (b) a transversion from G to T in exon 39: c.5779G>T; p.(Val1927Leu) that leads to a new missense substitution in a highly conserved amino acid Val1927 (Figure 2b,c). This novel variant, previously reported neither in HGMD nor Clinvar nor GnomAD, is located one nucleotide upstream of the canonical splicing acceptor site. The variant was predicted to have a deleterious effect (Align GV‐GD: Class C25, SIFT: Deleterious, Mutation‐Taster: Disease causing) and to also affect the splicing process (a total decrease in the score of the natural acceptor site of 59.0%, MaxEnt: −41.6%, NNSPLICE: −76.4%, and Splice Site Finder:‐8.5%). Taking all the evidence together, we classified *ABCA12*: c.5779G>T; p. (Val19227Leu) as likely pathogenic according to ACMG guidelines (Richards et al., 2015).

Segregation analysis of the variants in the family shows that the father of the patient is carrier of the *ABCA12 *c.6610C>T mutation, and the mother of the c.5779G>T mutation. None of the sisters are the carriers of any of these variants (Figure 2a).

## 4 DISCUSSION

Pathogenic variants in *ABCA12* have been described in ARCI including HI, CIE, and LI. HI shows the most severe phenotype, associated exclusively with *ABCA12* mutations. Homozygous or compound heterozygous missense *ABCA12* variants are frequently linked to LI and to a lesser extent CIE, whereas the majority of pathogenic variants associated with HI are homozygous or compound heterozygous nonsense and frameshift substitutions. Missense variants in combination with truncating mutations, including splice‐site variants, can be found in both CIE and HI (Akiyama, 2010). In this report we describe an Ecuadorian HI patient who harbors two different types of mutations in *ABCA12*. One is a nonsense variant which creates a premature codon. The second variant leads to a missense substitution in a conserved residue of the protein that is predicted to alter the splicing process by different algorithms. As both missense and splice‐site variants could lead to HI, any of these mechanisms could be affecting the pathogenicity of the variant. To better understand the implication of these type of variants in the development of the different ARCI subtypes, we performed a literature review of all *ABCA12* missense and splice‐site mutation carrier patients and their associated phenotypes. Thirty patients carrying *ABCA12* splice‐site variants were found (Table 1). Seven are homozygous carriers, and from these, those with pathogenic variants affecting the consensus splice‐sites and its surroundings, are classified as HI (patients 7, 11, 12, 18, 25, and 27) (Akiyama et al., 2005; Goldsmith et al., 2013; Hellström‐Pigg et al., 2016; Kelsell et al., 2005; Sheth, Bhavsar, Patel, Joshi, & Sheth, 2018; Thomas et al., 2008). Only two HI patients were compound heterozygous carriers of two different splice‐site variants (patients 24 and 28) (Esperón‐Moldes et al., 2018; Washio et al., 2017). Interestingly, the homozygous carrier of the synonymous variant c.3456G>A, p.(Ser1152=) (patient 10), located in the middle of the exon 24, shows a CIE phenotype. This milder phenotype could be explained by the fact that this mutation does not alter a consensus site but deregulates the expression of common transcripts; in this case a decrease in the expression of the wild type transcript and an increase in one minor transcript is observed (Goldsmith et al., 2013). Ten out of the 30 patients were compound heterozygous carriers of one *ABCA12* splice‐site variant affecting the consensus splice‐site and a second truncating variant including eight nonsense (patients 1, 4, 8, 9, 13, 17, 22, and 30) and two frameshift (patients 5 and 23); (Akiyama et al., 2005, 2007; Diociaiuti et al., 2016; Hellström‐Pigg et al., 2016; Kelsell et al., 2005; Loo, Batilando, Tan, & Koh, 2018; Scott et al., 2013; Takeichi, Sugiura, Matsuda, Kono, & Akiyama, 2013; Thomas et al., 2006; Tourette et al., 2012; Umemoto et al., 2011) all these patients were diagnosed with HI at birth (with exception of patient 13 of whom there was not available phenotypic information). However, there are still few data of patients carrying a combination of splice‐site and missense variants; from the eight patients reported to date, four showed CIE (patients 3, 15, 19, 26) (Bochner et al., 2017; Esperón‐Moldes et al., 2018; Fukuda et al., 2012), and one presented HI (patient‐16) (Hellström‐Pigg et al., 2016).

**Table 1:** ARCI splice‐site variant carrier patients described to date and bioinformatic prediction of variant outcomes

Patient	Splice‐site mutation	Location/predicted splicing defect	Splicing prediction scores^a^			Status	Second mutation	Phenotype	Ethnicity	Sex	Observations	Reference
			MaxEnt	NNSPLICE	SSF							
1	c.1062–3_1074del; p.(Leu355Lysfs*12)	Acceptor splice site of exon 10 (skip exon 10:−100%)	−100.0%	−100.0%	−100.0%	het	c.5005C>T; p.(Gln1669*)	HI	Japanese	Female	Systemic retinoids from postnatal day 6. Skin dramatically improved during infancy	Takeichi et al. (2013)
2	c.1287 + 2 T>G	Donor splice site of intron 11(skip of exon 11:−100%)	−100.0%	−100.0%	−100.0%	het	c.4139A>G; p.(Asn1380Ser)	Not specified	Spanish	Not reported	–	Esperón‐Moldes et al. (2018)
3	c.1287 + 2 T>G	Donor splice site of intron 11(skip of exon 11:−100%)	−100.0%	−100.0%	−100.0%	het	c.4139A>G; p.(Asn1380Ser)	CIE	Spanish	Male	Patient presented with small and whitish scales, erythroderma, and palmoplantar keratoderma	Esperón‐Moldes et al. (2018)
4	c.1782G>A; p.(Glu594=)	Exonic substitution exon 14 (change at donor site: −95.2%)	−100.0%	−90.3%	15.7%	het	c.596G>A, p.(Trp199*)	HI‐like	Scandinavian	No reported	Patient presented with collodion membrane at birth, ectropion, anhidrosis, and palmoplantar keratoderma	Hellström‐Pigg et al. (2016)
5	c.2332 + 2 T>G	Donor splice site of intron 17 (skip exon 17:−100%)	−100.0%	−100.0%	−100.0%	het	Exon 8 deletion	HI	British	Female	Neonatal mild hypothermia. Treated with systemic retinoids. Alive at 4 years of age	Kelsell et al. (2005)***; ***Scott et al. (2013)***; ***Thomas et al. (2006)
6	c.3295–1G>A	Acceptor splice site of intron 23 (skip exon 24:−100%)	−100.0%	−100.0%	−100.0%	het	unknown	HI	Malaysian	No reported	–	Numata et al. (2015)
7	c.3295–2A>G	Acceptor splice site intron 23(skip exon 24:−100%)	−100.0%	−100.0%	−100.0%	hom	–	HI	Japanese	Male	Survived infancy. Alive at publication (expresses some mutated ABCA12 protein)	Akiyama et al. (2005)
8	c.3295–2A>G	Acceptor splice site intron 23 (skip exon 24:−100%)	−100.0%	−100.0%	−100.0%	het	c.5848C>T, p.(Arg1950*)	HI	Japanese	Male	Died 3 days after birth	Akiyama et al. (2005)
9	c.3295–2A>G	Acceptor splice site intron 23(skip exon 24:−100%)	−100.0%	−100.0%	−100.0%	het	c.4543C>T; p.(Arg1515*)	HI	Japanese	Female	Systemic retinoids. Improved clinical symptoms at the age of 1 year and 7 months	Umemoto et al. (2011)
10	c.3456G>A; p.(Ser1152=)	Exonic substitution exon 24 (creates a novel acceptor splice site with similar scores as native site)	–	–	–	hom	–	CIE	Arab muslims	Female	Closely related parents. Several additional members of the family with similar condition	Goldsmith et al. (2013)
11	c.3829 + 1G>A	Donor splice site intron 26 (skip of exon 26:−100%)	−100.0%	−100.0%	−100.0%	hom	–	HI	unknown ethnicity	Not reported	–	Thomas et al. (2008)
12	c.3829 + 1G>A	Donor splice site intron 26 (skip of exon 26:−100%)	−100.0%	−100.0%	−100.0%	hom	–	HI	Scandinavian	Not reported	Patient presented with collodion membrane at birth, ectropion, anhidrosis, and palmoplantar keratoderma	Hellström‐Pigg et al. (2016)
13	c.4579 + 5G>A	Substitution in intron 30 (Change at donor site: −58.5%)	−67.2%	−49.9%	−16.0%	het	c.459 T>G, p.(Tyr153*)	ARCI	Italian	Female	Alive (6 years‐old) at examination	Diociaiuti et al. (2016)
14	c.5125_5128del; p.(Asp1709Thrfs*4)	Exonic deletion exon 33 (skip of exon 33:−100%)	−100.0%	−100.0%	−100.0%	het	unknown	HI	Syrian	Not reported	–	Thomas et al. (2008)
15	c.5128 + 3A>G	Substitution in intron 33 (change at donor site:–80.5%)	−74.3%	−86.7%	−5.8%	het	c.4139A>G, p.(Asn1380 Ser)	CIE	Japanese	Male	Alive (4 months) at publication	Fukuda et al. (2012)
16	c.5128 + 3A>G	Substitution in intron 33 (change at donor site:–80.5%)	−74.3%	−86.7%	−13.4%	het	c.3265G>T, p.(Val1089Phe)	HI	Scandinavian	Not reported	Patient presented with collodion membrane at birth, ectropion, anhidrosis, and palmoplantar keratoderma	Hellström‐Pigg et al. (2016)
17	c.5129–1G>T	Acceptor splice site of intron 33 (skip of exon 34:−100%)	−100.0%	−100.0%	−100.0%	het	c.7444C>T, p.(Arg2482*)	Harlequin fetus	French	Female	The fetus died at 31 weeks and 5 days gestation	Tourette et al. (2012)
18	c.5381 + 3_5381+4del	Deletion close to the donor splice site of exon 34 (skip of exon 34:−100.0%)	−100.0%	−100.0%	−36.6%	hom	–	HI	Irish/Polish mother Italian/German father	Not reported	–	Thomas et al. (2008)
19	c.5381 + 5G>A	Substitution in intron 34 near donor consensus (change at donor site: −95.5%)	−100.0%	−91.0%	−16.5%	het	c.4139A>G; p.(Asn1380Ser)	CIE	Spanish	Male	8 months old baby with small and whitish scales on an erythrodermic background	Esperón‐Moldes et al. (2018)
20	c.5690G>C; p.(Arg1897Thr)	Exonic substitution exon 37 (change at donor site:−85.3%)	−100.0%	−70.6%	−16.2%	het	unknown	HI	Eritrean/Jamaican	No reported	–	Thomas et al. (2008)
21^b^	c.5778 + 2 T>C	Donor splice site of intron 38 (skip of exon 38:−100%)	−100.0%	−100.0%	−0.5%	het	c.2956C>T, p.(Arg986Trp)	Not specified	Palestinian Armenian and Palestinian Catholic	Male	The child presented congenital exfoliative erythroderma, hypotrichosis, severe nail dystrophy, and failure to thrive	Bochner et al. (2017)
22	c.5779G>T; p.(Val1927Leu)	Exonic substitution exon 39 (change at acceptor site: −59.0%)	−41.6%	−76.4%	−8.5%	het	c. 6610C>T, p.(Arg2204*)	HI	Ecuadorian	Male	Alive (4 years old) at publication	This report
23	c.5884G>A; p.(Gly1962Ser)	Exonic substitution exon 39(change at donor site: −99%)	−100.0%	−97.9%	−16.4%	het	c.6858del; p.(Phe2286Leufs*6)	HI	Chinese	Female	Alive (5 months old) at publication	Loo et al. (2018)
24	c.5884 + 4_5884+5 del	Deletion close to the donor splice site of exon 39 (skip of exon 39: −69.2%)	−55.5%	−83.0%	−7.2%	het	c.7239G>A; p.(Leu2413=)	HI	Japanese	Male	Alive (2 years old) at publication	Washio et al. (2017)
25	c.5939 + 4A>G	Substitution in intron 40, near donor consensus (change at donor site: −49.9%	−41.5%	−58.3%	−12.5%	hom	–	HI	Gujarati, Indian	Female	Alive newborn at examination. She succumbed to septicemia 4 days after birth	Sheth et al., (2018)
26	c.5940–1G>C	Acceptor splice site of intron 40 (skip of exon 41: −100.0%)	−100.0%	−100.0%	−100.0%	het	c.2956C>T, p.(Arg986Trp)	CIE	Japanese	Female	Alive (9 years old) at publication. Younger sister suffered from severe skin symptoms, complications, and died	Fukuda et al. (2012)
27	c.6233 + 1G>T	Donor splice site intron 42 (skip of exon 42: −100.0%)	−100.0%	−100.0%	−100.0%	hom	–	HI	Iranian	Not reported	Septicemia. Died at age 4 months	Kelsell et al. (2005)
28	c.6394–2A>G	Acceptor splice site of intron 43 (skip of exon 44:−100%)	−100.0%	−100.0%	−100.0%	het	c.7436G>A; p.(Arg2479Lys)	HI	Spanish	Female	Alive (9 years old) at examination. The patient shows a CIE phenotype.	Esperón‐Moldes et al. (2018)
29	c.7105–22_7105–4 del	Deletion in intron 47 close to the acceptor splice site of exon 48 (skip of exon 48:−100%).	−100.0%	−99.9%	−100.0%	het	c.6941 T>C; p.(Ile2314Thr)	Not specified	Spanish	Not reported	–	Esperón‐Moldes et al. (2018)
24	c.7239G>A; p.(Leu2413=)	Exonic substitution exon 48 (change at donor site: −51.2%)	−44.9%	−57.4%	−14.1%	het	c.5884 + 4_5884+5del	HI	Japanese	Male	Alive (2 years old) at publication	Washio et al. (2017)
30	c.7436G>A; p.(Arg2479Lys)	Exonic substitution exon 50 (change at donor site: −99.5%)	−100.0%	−99.1%	−15.5%	het	c.3746C>A, p.(Ser1249*)	HI	French	Male	Died soon after birth	Akiyama et al. (2007)
28	c.7436G>A; p.(Arg2479Lys)	Exonic substitution exon 50 (change at donor site: −99.5%)	−100.0%	−99.1%	−15.5%	het	c.6394–2A>G	HI	Spanish	Female	Alive (9 years old) at examination. The patient shows a CIE phenotype.	Esperón‐Moldes et al. (2018)

Note

GenBank reference sequence (NM 173076.2, NP_775099)

ARCI: autosomal recessive congenital ichthyosis; CIE: congenital ichthyosiform erythroderma; het: heterozygous; HI: harlequin ichthyosis; HI‐like: CIE patients with ultrastructural findings resembling those detected in previous HI case; hom: homozygous; SSF: Splice Site Finder.

a Percentages of variation predicted by Alamut at consensus splice‐sites.

b Note that this patient shows an atypical ARCI phenotype (including severe hair and nail manifestations) and he also carries two additional heterozygous mutations in the *CAPN12* gene [c.1511C>A; p.(P504Q), c.1090_1129del; p.(Val364Lysfs*11)].

We also identified a total of sixty‐three *ABCA12 *missense carrier patients. As shown in Table 2, most of HI patients bear at least one truncating variant in one of the two alleles (Patients 32, 34, 35, 41, 42, 50, 67, 79, 80, 81, 83, 86, 89, 91–93) (Akiyama et al., 2006, 2007; Esperón‐Moldes et al., 2018; Hellström‐Pigg et al., 2016; Kelsell et al., 2005; Loo et al., 2018; Numata et al., 2015; Peterson, Lofgren, Bremmer, & Krol, 2013; Scott et al., 2013; Tanahashi, Sugiura, Sato, & Akiyama, 2016; Xie et al., 2016). Two HI patients were described as carriers of missense variants in both alleles (Patients 31 and 47), however, the variants identified in patient 31; *ABCA12:* c.130C>G; p.(Arg44Gly) and c.2033A>G p.(Asn678Ser) (Scott et al., 2013) could be not the causative variants assuming that almost all algorithms predict a non‐deleterious effect and considering that a heterozygous known *TGM1* mutation: c.401A>G; p.(Tyr134Cys) was also detected in this same patient; in the case of patient 47, described as carrier of a pathogenic variant c.3535G>A; p.(Gly1179Arg) in homozygous state, the zigosity needs to be confirmed. Interestingly, we did not find any difference between the type of mutations in patients with moderate and severe HI phenotypes. As previously reported, CIE patients carry at least one missense variant in combination with other missense, nonsense, splice‐site and frameshift mutations, while almost all LI patients are carriers of missense mutations in both alleles. Exceptions are two LI cases (patients 57 and 64) who harbor nonsense and frameshift variants. Interestingly these two patients did not show a more severe phenotype compared to other LI patients who carried missense mutations in both alleles (Akiyama et al., 2008; Bučková et al., 2016; Chao, Aleshin, Goldstein, Worswick, & Hogeling, 2018; Esperón‐Moldes et al., 2018; Fukuda et al., 2012; Hellström‐Pigg et al., 2016; Israeli et al., 2013; Lefèvre et al., 2003; Loo et al., 2018; Murase et al., 2018; Natsuga et al., 2007; Nawaz et al., 2012; Numata et al., 2015; Sakai et al., 2009; Scott et al., 2013; Shimizu et al., 2013; Sitek et al., 2018; Thomas et al., 2008; Wada et al., 2017; Wakil et al., 2016). The majority of the genotype‐phenotype associations found in these patients are in accordance with the correlations previously established by Akiyama, with some few exceptions as previously stated (Akiyama, 2010).

**Table 2:** ARCI missense variant carrier patients described to date and bioinformatic prediction of variant outcomes

Patient	Missense mutation	Location in the protein	Predicted splicing defect	Missense prediction scores			Status	Second mutation	Phenotype	Ethnicity	Sex	Observations	Reference
				Align GV‐GD^a^	SIFT	Mutation taster							
31	c.130C>G; p.(Arg44Gly)	–	None	C0	D	P	het	c.2033A>G; p.(Asn678Ser)	HI	unknown ethnicity	Not reported	Mild HI phenotype. This patient also carries the *TGM1 *c.401A>G mutation	Scott et al. (2013)
32	c.179G>C; p.(Arg60Pro)	–	None	C0	D	DC	het	c.1300C>T; p.(Arg434*)	HI	unknown ethnicity	Female	–	Scott et al. (2013)
33	c.1033A>C; p.(Thr345Pro)	–	None	C0	T	P	hom	–	CIE	Japanese	Female	A 37‐year‐old woman with CIE accompanied by malignant melanoma	Natsuga et al. (2007)
34	c.1160G>A; p.(Ser387Asn)	–	None	C0	T	P	het	c.4158_4160del; p.(Thr1387del)	HI	Japanese	Male	Moderate clinical severity	Akiyama et al. (2006
35	c.1446A>C; p.(Glu482Asp)	–	None	C0	T	P	het	c.7444C>T; p.(Arg2482*)	HI	unknown ethnicity	Not reported	–	Scott et al., 2013
31	c.2033A>G; p.(Asn678Ser)	–	None	C0	T	P	het	c.130C>G; p.(Arg44Gly)	HI	unknown ethnicity	Not reported	Mild HI phenotype. This patient also carries the *TGM1 *c.401A>G mutation	Scott et al., 2013
36	c.2634C>G; p.(Phe878Leu)	–	None	C0	T	DC	het	c.4139A>G; p.(Asn1380Ser)	CIE	Czech	Not reported	Fine, whitish scales, and generalized erythema	Bučková et al., 2016
37	c.2638G>C; p.(Val880Leu)	–	None	C0	D	DC	het	c.3673C>T; p.(Arg1225*)	ARCI	Caucasian	Female	69 years old at the moment of study	Sitek et al. (2018)
38	c.2956C>T; p.(Arg986Trp)	–	None	C65	D	DC	het	c.5940–1G>C	CIE	Japanese	Female	9‐year‐old girl with generalized scales on an erythrodermic skin, mild ectropion, alopecia, and mild auricular malformation	Fukuda et al. (2012)***; *** Numata et al. (2015)
39	c.2956C>T; p.(Arg986Trp)	–	None	C45	D	DC	hom	–	CIE	Japanese	Not reported	–	Numata et al. (2015)
40	c.2956C>T, p.(Arg986Trp)	–	None	C65	D	DC	het	c.5778 + 2 T>C	ARCI	Palestinian Armenian and Palestinian Catholic	Male	CEE, hypotrichosis, severe nail dystrophy, FTT	Bochner et al. (2017)
41	c.3085G>A; p.(Glu1029Lys)	–	None	C55	D	DC	het	c.859C>T; p.(Arg287*)	HI	Chinese	Not reported	–	Numata et al. (2015)
42	c.3265G>T; p.(Val1089Phe)	–	None	C45	D	DC	het	c.5128 + 3A>G	HI	Scandinavian	Not reported	Patient presented with collodion membrane at birth, ectropion, anhidrosis, and PPK	Hellström‐Pigg et al. (2016)
43	c.3299 T>G; p.(Met1100Arg)	–	Predicted change at acceptor site 5 bps upstream: +0.5%	C0	D	DC	het	c.7164dup; p.(Met2389Tyrfs*27)	CIE/HI	unknown ethnicity	Female	Intermediate phenotype between HI and CIE	Peterson et al. (2013)
44	c.3407G>A; p. p.(Gly1136Asp)	–	None	C0	D	DC	het	c.5005C>T; p.(Gln1669*)	CIE	Japanese	Male	Fine, whitish scales on hyperkeratotic, erythrodermic skin, mild ectropion, and eclabium	Akiyama et al. (2008)
45	c.3470C>T; p.(Ser1157Leu)	TNM	None	C15	D	DC	hom	–	LI	Saudi	–	Four affected members in the same family. They all showed PPK	Wakil et al. (2016)
46	c.3470C>T; p.(Ser1157Leu)	TNM	None	C15	D	DC	het	unknown	CIE	Japanese	Not reported	–	Numata et al. (2015)
47	c.3535G>A; p.(Gly1179Arg)	TNM	None	C65	D	DC	hom	–	HI	Hmong/Laotian	Not reported	Sepsis, FTT, corneal perforation, respiratory failure, developmental delay	Thomas et al. (2006)
48	c.3704G>C; p.(Trp1235Ser)	–	None	C65	D	DC	het	c.5848C>T; p.(Arg1950*)	CIE	Japanese	Male	6 years at the moment of the study	Sakai et al. (2009)
36	c.4139A>G; p.(Asn1380Ser)	NBF1	None	C0	D	DC	het	c.2634C>G; p.(Phe878Leu)	CIE	Czech	Not reported	Fine, whitish scales, and generalized erythema	Bučková et al., 2016
49	c.4139A>G; p.(Asn1380Ser)	NBF1	None	C45	D	DC	het	c.5128 + 3A>G	CIE	Japanese	Male	Male born as a collodion baby, with whitish scales and generalized erythrodermic skin	Fukuda et al. (2012)**; **Numata et al. (2015)
50	c.4139A>G; p.(Asn1380Ser)	NBF1	None	C45	D	DC	het	c.4554G>A; p.(Trp1518*)	HI	Scandinavian	Not reported	Collodion membrane, ectropion, anhidrosis, and PPK	Hellström‐Pigg et al. (2016)
51	c.4139A>G; p.(Asn1380Ser)	NBF1	None	C45	D	DC	hom	–	LI	Moroccan	Not reported	Collodion membrane, large dark scales, ectropion, and PPK	Lefèvre et al. (2003)
52	c.4139A>G; p.(Asn1380Ser)	NBF1	None	C45	D	DC	het	c.4951G>A; p.(Gly1651Ser)	LI	Algeria	Not reported	Collodion membrane, large dark scales, ectropion, and PPK	Lefèvre et al. (2003)
53	c.4139A>G; p.(Asn1380Ser)	NBF1	None	C45	D	DC	hom	–	LI	Algeria	Not reported	Collodion membrane, large dark scales, ectropion, and PPK	Lefèvre et al. (2003)
54	c.4139A>G; p.(Asn1380Ser)	NBF1	None	C45	D	DC	het	c.4070C>A; p.(Ser1357*)	CIE/LI	unknown ethnicity	Female	–	Scott et al. (2013)
55	c.4139A>G; p.(Asn1380Ser)	NBF1	None	C45	D	DC	het	c.1287 + 2 T>G	CIE	Spanish	Male	Small, whitish scales with eythroderma, PPK, PH, and altered sweating	Esperón‐Moldes et al. (2018)
56	c.4139A>G; p.(Asn1380Ser)	NBF1	None	C45	D	DC	hom	–	CIE	Spanish	Female	Small, dark scales with alopecia and PPK	Esperón‐Moldes et al. (2018)
57	c.4139A>G; p.(Asn1380Ser)	NBF1	None	C45	D	DC	het	c.3837_3838del; p.(Tyr1279*)	LI	Spanish	Female	Small, whitish scales with ectropion, alopecia, and PPK	Esperón‐Moldes et al. (2018)
58	c.4139A>G; p.(Asn1380Ser)	NBF1	None	C45	D	DC	hom	–	CIE	Spanish	Female	Small, whitish scales with eythroderma, collodion membrane, PPK, PH, and altered sweating	Esperón‐Moldes et al. (2018)
59	c.4139A>G; p.(Asn1380Ser)	NBF1	None	C45	D	DC	het	c.1287 + 2 T>G	Not specified	Spanish	Not reported	–	Esperón‐Moldes et al. (2018)
60	c.4139A>G; p.(Asn1380Ser)	NBF1	None	C45	D	DC	het	c.178C>T; p.(Arg60*)	CIE	Spanish	Female	Big, whitish scales with eythroderma, collodion membrane, alopecia, ectropion, PPK, PH, and altered sweating	Esperón‐Moldes et al. (2018)
61	c.4139A>G; p.(Asn1380Ser)	NBF1	None	C45	D	DC	het	c.5381 + 5G>A	CIE	Spanish	Male	Small, whitish scales with eythroderma, collodion membrane, PPK, PH, and altered sweating	Esperón‐Moldes et al. (2018)
62	c.4139A>G; p.(Asn1380Ser)	NBF1	None	C45	D	DC	het	c.5641C>T; p.(Arg1881*)	CIE	Spanish	Male	Small, whitish scales with alopecia, ectropion, eythroderma, PPK, PH, and altered sweating	Esperón‐Moldes et al. (2018)
63	c.4139A>G; p.(Asn1380Ser)	NBF1	None	C45	D	DC	het	c.6031del; p.(Glu2011Asnfs*17)	CIE	Japanese	Female	At birth, entire body surface covered with thick, gray scales on a background of erythrodermic skin	Murase et al. (2018)
64	c.4139A>G; p.(Asn1380Ser)	NBF1	None	C45	D	DC	het	c.4491_4493del3ins22	LI	unknown ethnicity	Female	Large, brown plte‐like hyperkeratotic scales, PPK, and hyperlinearity of the trunk	Chao et al. (2018)
65	c.4142G>A; p.(Gly1381Glu)	NBF1	None	C65	D	DC	hom	–	LI	Morocco	Not reported	Collodion membrane, large dark scales, ectropion, and PPK	Lefèvre et al. (2003)
66	c.4481 T>C; p.(Ile1494Thr)	NBF1	None	C25	D	DC	het	Unknown	CIE	Japanese	Male	42‐year‐old man with CIE and cutaneous squamous cell carcinoma	Natsuga et al. (2007)
67	c.4541G>A; p.(Arg1514His)	NBF1	None	C0	D	DC	het	c.4896del; p.(Ser1633Hisfs*30)	HI‐like	Scandinavian	Not reported	Collodion membrane, ectropion, anhidrosis, and PPK	Hellström‐Pigg et al. (2016)
68	c.4541G>A; p.(Arg1514His)	NBF1	None	C0	D	DC	hom	–	CIE	Japanese	Male	52 years at the moment of the study	Sakai et al. (2009)
69	c.4541G>A; p.(Arg1514His)	NBF1	None	C0	D	DC	hom	–	LI	Mali	Not reported	Collodion membrane, large dark scales, ectropion, and PPK	Lefèvre et al. (2003)
70	c.4544G>A; p.(Arg1515Gln)	NBF1	None	C0	D	DC	het	c.4553G>A; p.(Trp1518*)	CIE	Jewish	Not reported	–	Israeli et al. (2013)
71	c.4615G>A; p.(Glu1539Lys)	NBF1	None	C55	D	DC	hom	–	LI	Algeria	Not reported	Milder form of ichthyosis with smaller and whitish scales	Lefèvre et al. (2003)
72	c.4676G>T; p.(Gly1559Val)	–	None	C65	D	DC	hom	–	CIE	Pakistani	Not reported	Five affected members with small, fine scales, erythroderma, PPK, and mild ectropion. Legs showed brownish scales similar to LI	Nawaz et al. (2012)
73	c.4676G>T; p.Gly1559Val	–	None	C65	D	DC	hom	–	ARCI	Pakistani	Female	26 years old at the moment of study	Sitek et al. (2018)
74	c.4723A>C; p.(Thr1575Pro)	–	None	C0	D	DC	het	c.6031del; p.(Glu2011Asnfs*17)	CIE	Japanese	Female	3‐year‐old girl with generalised scales, erythroderma, ectropion, eclabium, severely deformed ears, and alopecia	Fukuda et al. (2012)**; **Numata et al. (2015)
75	c.4723A>C; p.(Thr1575Pro)	–	None	C0	D	DC	het	c.4951G>A; p.(Gly1651Ser)	CIE	Japanese	Male	3‐month‐old boy born as a collodion baby, with generalized whitish scales on a erythrodermic skin	Fukuda et al. (2012)**; **Numata et al. (2015)
75	c.4951G>A; p.(Gly1651Ser)	–	None	C55	D	DC	het	c.4723A>C; p.(Thr1575Pro)	CIE	Japanese	Male	3‐month‐old boy born as a collodion baby, with generalized whitish scales on a erythrodermic skin	Fukuda et al. (2012)**; **Numata et al. (2015)
76	c.4951G>A; p.(Gly1651Ser)	–	None	C55	D	DC	hom	–	LI	Algeria	Not reported	Collodion membrane, large dark scales, ectropion, and PPK	Lefèvre et al. (2003)
52	c.4951G>A; p.(Gly1651Ser)	–	None	C55	D	DC	het	c.4139A>G; p.(Asn1380Ser)	LI	Algeria	Not reported	Collodion membrane, large dark scales, ectropion, and PPK	Lefèvre et al. (2003)
77	c.5393C>T; p.(Pro1798Leu)	–	None	C0	D	DC	het	unknown	CIE	Japanese	Female	Less than one year at the moment of the study	Sakai et al. (2009)
78	c.5690G>C; p.(Arg1897Thr)	–	Exonic substitution exon 37 (change at donor site:−85.3%)	C65	D	DC	het	unknown	HI	Eritrean/Jamaican	Not reported	–	Thomas et al. (2008)
79	c.5779G>T; p.(Val1927Leu)	–	Exonic substitution exon 39 (change at acceptor site: −59.0%)	C25	D	DC	het	c.6610C>T, p.(Arg2204*)	HI	Ecuadorian	Male	Alive (4‐year‐old) at publication	This report
80	c.5884G>A; p.(Gly1962Ser)	–	Exonic substitution exon 39(change at donor site: −99%)	C55	D	DC	het	c.6858del; p.(Phe2286Leufs*6)	HI	Chinese	Female	5 months at publication. Severe HI phenotype.	Loo et al. (2018)
81	c.5936C>G; p.(Ala1979Gly)	–	None	C0	D	DC	het	c.6858del; p.(Phe2286Leufs*6)	HI atypical	unknown ethnicity	Male	HI atypical, chrysalis	Scott et al. (2013)
82	c.5939C>A; p.(Thr1980Lys)	–	None	C0	D	DC	het	unknown	CIE	Japanese	Female	One year at the moment of the study	Sakai et al. (2009)
83	c.5985G>A; p.(Met1995Ile)	TNM	Novel acceptor splice site with similar scores as native site	C0	T	DC	het	c.1194_1221del; p.(Gln400Phefs*18)	HI	Japanese	Female	2.5 years old at publication, clinical features typical of HI	Tanahashi et al. (2016)
84	c.6263 T>C; p.(Leu2088Pro)	TNM	None	C65	D	DC	het	c.1002_1004delinsT; p.(Thr335Alafs*5)	CIE	Scandinavian	Not reported	–	Hellström‐Pigg et al. (2016)
85	c.6431 T>C; p.(Phe2144Ser)	–	None	C65	D	DC	het	c.4139A>G; p.(Asn1380Ser)	CIE	Japanese	Female	A 5‐year‐old girl born as a collodion baby. Clinical features typical of CIE	Shimizu et al. (2013)
86	c.6443C>A; p.(Pro2148Gln)	–	None	C65	D	DC	het	c.5232G>A; p.(Trp1744*)	HI	Chinese	Female	Typical HI fetus terminated with two more cases in the family	Xie et al. (2016)
87	c.6551A>T; p.(Asn2184Ile)	–	None	C55	D	P	het	c.6696_6699dup; p.(Asp2234*)	CIE	Japanese	Female	Mild CIE with periodic exacerbation	Wada et al. (2017)
88	c.6900C>A; p.(Phe2300Leu)	NBF2	None	C15	D	DC	hom	–	LI	Saudi	Not reported	Large scales with erythroderma and keratoderma.	Wakil et al. (2016)
89	c.7093G>A; p.(Asp2365Asn)	NBF2	None	C0	D	P	het	c.5229del; p.(Trp1744Glyfs*24)	HI	Italian	Not reported	6 years old at publication, nystagmus, PDA, neonatal sepsis	Kelsell et al. (2005)
90	c.7187G>C; p.(Arg2396Thr)	NBF2	None	C65	D	DC	het	c.986–719_1061+1902del; p.(Asp330Serfs*2)	ARCI	Caucasian	Male	Less than one year old at the moment of study. Osteopenia	Sitek et al. (2018)
91	c.7412G>A; p.(Gly2471Glu)	–	None	C65	D	DC	het	c.7137del; p.(Met2380Cysfs*25)	HI‐like	Scandinavian	Not reported	Collodion membrane, ectropion, anhidrosis, and PPK	Hellström‐Pigg et al. (2016)
92	c.7436G>A; p.(Arg2479Lys)	–	Exonic substitution exon 50 (change at donor site: −99.5%)	C25	D	DC	het	c.3746C>A, p.(Ser1249*)	HI	French	Male	Died soon after birth	Akiyama et al. (2007)
93	c.7436G>A; p.(Arg2479Lys)	–	Exonic substitution exon 50 (change at donor site: −99.5%)	C25	D	DC	het	c.6394–2A>G	HI	Spanish	Female	Alive (9 years old) at examination. The patient now shows a CIE phenotype.	Esperón‐Moldes et al. (2018)

Note

GenBank reference sequence (NM 173,076.2, NP_775099)

ARCI: autosomal recessive congenital ichthyosis; CEE: congenital exfoliative erythroderma; CIE: congenital ichthyosiform erythroderma; D: deleterious; DC: disease‐causing; FTT: failure to thrive; het: heterozygous; HI: harlequin ichthyosis; HI‐like: CIE patients with ultrastructural findings resembling those detected in previous HI cases; hom: homozygous; LI: lamellar ichthyosis; P: polymorphism; PDA: patent ductus arteriosus; PH: palmar hiperlinearity; PPK: palmoplantar keratoderma; T: tolerated.

a Align GV‐GD prediction classes form a spectrum (C0, C15, C25, C35, C45, C55, C65) with C65 most likely to interfere with function and C0 least likely.

Given the current available data, further characterization of missense variants, including the confirmation of the zigosity in putative homozygous patients and the assessment of their impact in the splicing process, would be needed to better elucidate this genotype‐phenotype correlation.

In brief, our case expands the spectrum of *ABCA12* reported disease‐causing variants. Additionally the literature review of splice‐site and missense *ABCA12* mutations performed in this study contributes to further understanding of the complex genotype‐phenotype correlations in the different subtypes of ARCI.

## CONFLICT OF INTEREST

The authors declare that they have no conflict of interest.
